# A case of inferior phrenic artery injury after chest drainage treated laparoscopically

**DOI:** 10.1016/j.tcr.2023.100763

**Published:** 2023-01-09

**Authors:** Yohei Sanmoto, Naoyuki Hanari, Shunji Kinuta

**Affiliations:** Department of Gastrointestinal Surgery, Takeda General Hospital, 3-27 Yamagachou, Aizuwakamatsu-shi, Fukushima 965-8585, Japan

**Keywords:** Hemostasis, Chest drainage, Laparoscopy, Vascular injury

## Abstract

Chest drainage is a commonly performed surgical procedure. However, caution is required when performing this procedure because of its serious complications. One complication is vascular injury. Endovascular treatment has been prioritized in patients requiring hemostasis. We report the case of an 87-year-old woman, who presented to our hospital with dyspnea caused by massive pleural effusion. We decided to perform chest tube placement for the purpose of diagnosis and treatment. The inferior phrenic artery was injured during chest drainage, resulting in hemorrhagic shock. Catheter embolization was considered, but it was deemed difficult due to the patient's abnormal blood vessel. Instead, hemostasis was induced via laparoscopy. This is the first report on the safety of laparoscopic hemostasis for inferior phrenic artery bleeding. By devising, we were able to perform hemostatsis safely under laparoscopy.

## Introduction

Chest drainage is a commonly performed surgical procedure. However, its complications include pneumothorax, hemothorax, vascular injury, and organ injury. We report a case of hemorrhagic shock, caused by an injury to the right inferior phrenic artery during chest tube placement. Hemostasis was successfully achieved laparoscopically.

## Case presentation

**Fig. 1:**
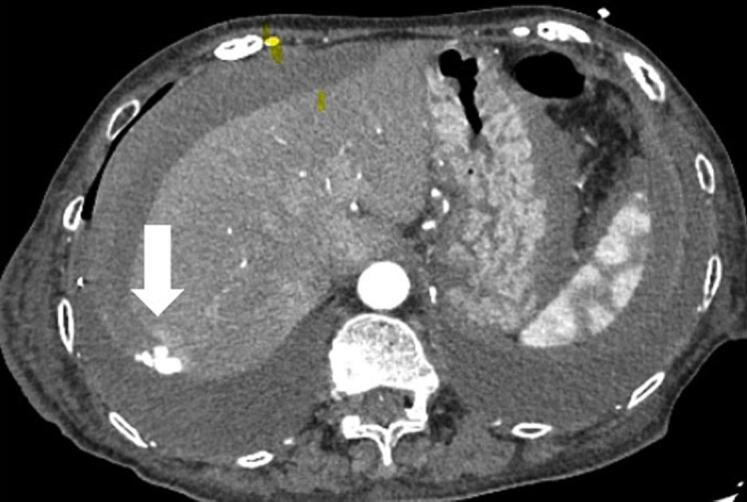
Computed tomography shows extravasation from the right inferior phrenic artery (arrow).

**Fig. 2:**
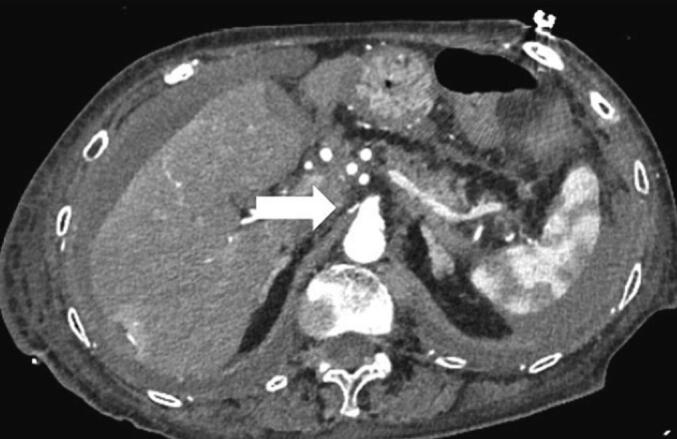
The right inferior phrenic artery (arrow) diverges sharply toward the dorsal side from the root of the celiac trunk.

**Fig. 3:**
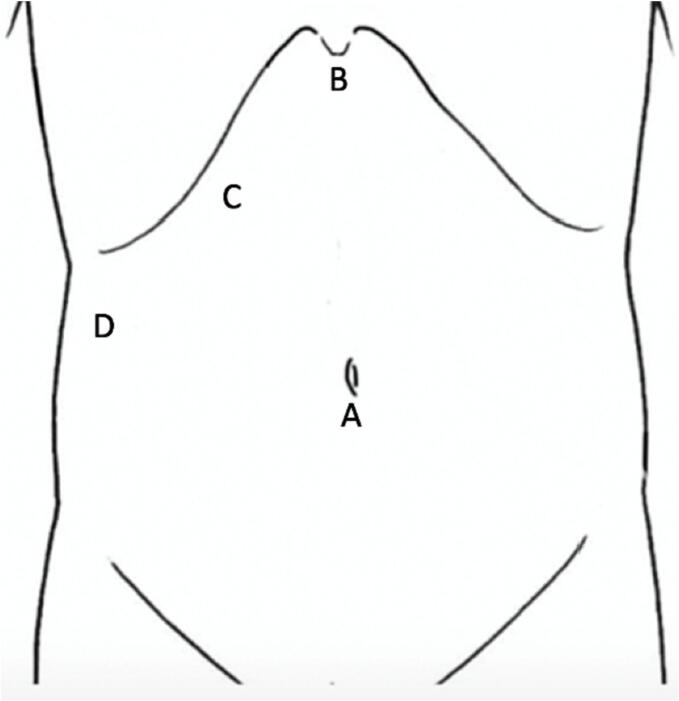
Layout of the ports. (A: 12 mm, B: 5 mm, C: 12 mm, D: 5 mm).

**Fig. 4:**
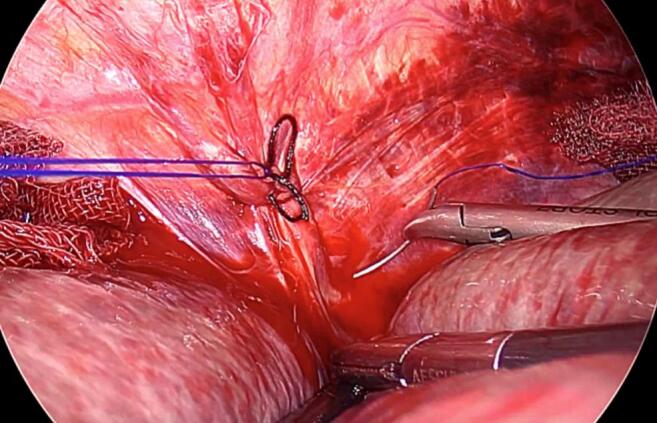
A gauze was used to secure a working space.

An 87-year-old woman consulted our hospital for treatment for dyspnea. Computed tomography showed a massive pleural effusion. Therefore, chest tube drainage was performed. Under ultrasonic guidance, a 12-Fr aspiration catheter was inserted into the right thoracic cavity, resulting in fresh blood drainage. The post-procedure radiograph revealed a tube in the right thoracic cavity with no pneumothorax. Three hours later, the patient reported sudden-onset discomfort and decreased blood pressure. Her blood tests showed decreased hemoglobin levels from 11.3 g/dL to 6.2 g/dL, and she was diagnosed with hemorrhagic shock. Computed tomography showed the drainage tube, inserted from the right ninth intercostal space, and active extravasation of the contrast medium from the right inferior phrenic artery into the abdominal cavity (Fig. 1). Angiography was considered to address the bleeding, but it was judged difficult because the right inferior phrenic artery diverged sharply toward the dorsal side from the root of the celiac artery (Fig. 2). Thus, laparoscopic hemostasis was performed. The surgery was initiated through four ports, including the umbilical region (Fig. 3). Upon inspecting the abdominal cavity, multiple blood clots were found, mainly in the upper abdomen. The bleeding site was identified while suctioning. The patient had arterial bleeding from the peripheral branch of the right inferior phrenic artery. Therefore, three needles were used for suturing, and hemostasis was performed using a 3-0 non-absorbable thread (Fig. 4). Although it was difficult to secure the visual field, a working space was obtained by inserting a sterilized gauze into the right liver dome to straddle the bleeding point and push down the liver. Since the distance between the port insertion and bleeding site was near, an extracorporeal ligation method was adopted to perform reliable ligation. After confirming the absence of persistent bleeding or organ damage, the drain was inserted, and the operation was completed. The operation time was 92 min. The postoperative course was uneventful without progression of anemia. Computed tomography on the third postoperative day showed no active bleeding, and the drain was removed. She resumed eating on the fourth postoperative day and was discharged 21 days after the operation.

## Discussion

Complications associated with chest drainage include injury to the intrathoracic organs, such as the lungs, diaphragm, heart, mediastinum, and esophagus; injury to the intraperitoneal organs, such as the stomach, liver, spleen, and gastrointestinal tract; and malposition of catheters [1]. In particular, diaphragmatic injury likely results from the drainage tube insertion in the inferior region of the intercostal space [1]. The inferior phrenic artery is a blood vessel that branches between the 12th thoracic vertebra and second lumbar vertebra from the aorta or celiac artery [2], running on the diaphragmatic surface. However, the inferior phrenic artery reportedly has various origins. Loukas et al. [3] reported that the origins of the right inferior phrenic artery were the celiac trunk (40 %), aorta (38 %), right renal artery (17 %), left gastric artery (3 %), and hepatic artery proper (1 %). Similarly, the left inferior phrenic artery originated from the celiac trunk (47 %), aorta (45 %), left renal artery (5 %), left gastric artery (2 %), and hepatic artery proper (1 %). There have been previous case reports on vascular injuries caused by blunt trauma [4], abdominal surgery [5], [6], and cardiopulmonary resuscitation [7]. Transcatheter embolization has been widely used to achieve hemostasis, but this approach is considered difficult based on the bifurcation site and angle of the vessel. After the patient in this case had been diagnosed, endovascular treatment was also considered because it was less invasive. However, the right inferior phrenic artery diverged at a sharp angle from the root of the celiac trunk. Thus, endovascular treatment was not indicated, and surgical treatment was selected. Laparoscopic surgery was chosen over open surgery because the injured site was deep in the dorsal side of the diaphragm. Laparoscopy offered the advantage of confirming bleeding points and suturing. By arranging port sites similar to laparoscopic cholecystectomy and inserting sterilized gauze in the liver dome, the liver was excluded. Moreover, a working space was secured, and hemostasis was successfully performed in a narrow space. A literature search on PubMed was conducted using the keywords, “inferior phrenic artery,” “injury,” and “laparoscopic.” However, similar reports were not found. To the best of our knowledge, this is the first report of laparoscopic hemostasis for inferior phrenic arterial bleeding. The laparoscopic approach is a helpful treatment option for cases wherein an endovascular treatment is deemed difficult.
